# Using MATLAB software with Tomcat server and Java platform for remote image analysis in pathology

**DOI:** 10.1186/1746-1596-6-S1-S18

**Published:** 2011-03-30

**Authors:** Tomasz Markiewicz

**Affiliations:** 1 Institute of Theory of Electrical Engineering, Measurement and Information System, Warsaw University of Technology, ul. Koszykowa 75, 00-662 Warsaw, Poland; 2 Department of Pathology, Military Institute of Medicine, ul. Szaserow 128, 04-141 Warsaw, Poland

## Abstract

**Background:**

The Matlab software is a one of the most advanced development tool for application in engineering practice. From our point of view the most important is the image processing toolbox, offering many built-in functions, including mathematical morphology, and implementation of a many artificial neural networks as AI. It is very popular platform for creation of the specialized program for image analysis, also in pathology. Based on the latest version of Matlab Builder Java toolbox, it is possible to create the software, serving as a remote system for image analysis in pathology via internet communication. The internet platform can be realized based on Java Servlet Pages with Tomcat server as servlet container.

**Methods:**

In presented software implementation we propose remote image analysis realized by Matlab algorithms. These algorithms can be compiled to executable *jar* file with the help of Matlab Builder Java toolbox. The Matlab function must be declared with the set of input data, output structure with numerical results and Matlab web figure. Any function prepared in that manner can be used as a Java function in Java Servlet Pages (JSP). The graphical user interface providing the input data and displaying the results (also in graphical form) must be implemented in JSP. Additionally the data storage to database can be implemented within algorithm written in Matlab with the help of Matlab Database Toolbox directly with the image processing. The complete JSP page can be run by Tomcat server.

**Results:**

The proposed tool for remote image analysis was tested on the Computerized Analysis of Medical Images (CAMI) software developed by author. The user provides image and case information (diagnosis, staining, image parameter etc.). When analysis is initialized, input data with image are sent to servlet on Tomcat. When analysis is done, client obtains the graphical results as an image with marked recognized cells and also the quantitative output. Additionally, the results are stored in a server database. The internet platform was tested on PC Intel Core2 Duo T9600 2.8GHz 4GB RAM server with 768x576 pixel size, 1.28Mb tiff format images reffering to meningioma tumour (x400, Ki-67/MIB-1). The time consumption was as following: at analysis by CAMI, locally on a server – 3.5 seconds, at remote analysis – 26 seconds, from which 22 seconds were used for data transfer via internet connection. At jpg format image (102 Kb) the consumption time was reduced to 14 seconds.

**Conclusions:**

The results have confirmed that designed remote platform can be useful for pathology image analysis. The time consumption is depended mainly on the image size and speed of the internet connections. The presented implementation can be used for many types of analysis at different staining, tissue, morphometry approaches, etc. The significant problem is the implementation of the JSP page in the multithread form, that can be used parallelly by many users. The presented platform for image analysis in pathology can be especially useful for small laboratory without its own image analysis system.

## Background

The image analysis in pathology is a very wide and differentiated topic focusing interests of many researchers [1-6]. The digital microscopic images in pathology are very different, according to the type of tissue, disease, used staining, and required type of analysis (e.g. quantitative, morphometric). Matlab software [7] is a one of the most developed tool for application in engineering practice and can be useful in this topic. Among many different technical aspects, it supports image processing, giving many built-in functions, especially directed to mathematical morphology and offering implementation of many artificial neural networks, that can be used together with morphological processing for recognition of the cells. Thanks to these features, Matlab is a popular platform for creation of specialized program for image analysis, also in pathology [8].

In the last years the Computerized Analysis of Medical Images (CAMI) software, written in Matlab, was designed and developed by us in the Military Institute of Medicine, Warsaw, Poland [9,10]. It was used in several research projects as a tool for automatic quantitative and morphometric image analysis. The main directions of application were breast cancer (ER/PR receptors), tumours of the nervous system (neuroblastoma, meningioma, oligodendroglioma – Ki-67 staining) and gastric inflammatory (chromogranin, serotonin and somatostatin antibodies). The positive results of CAMI application inclined us to idea to build internet platform offering the remote desktop to CAMI.

The latest version of Matlab offers tools for automatic building the executable files of our functions designed in Matlab code by using Java language. With the help of Matlab Builder Java toolbox [7] it is possible to build executable *jar* files for use by Java Servlet Pages (JSP) [11] in internet www server as a servlet (program executed on server). However example application (existing in internet and presented in the Mathworks web side [7]) restrict to using Matlab for numerical calculation only or sometimes with graphical visualization, completely omitting the problem of analysis of the images supplied by the remote user. Only one example software (HDRExplorer [12]) with the analysis of the image sequence can be found, but this system works only with the data files stored on the server, not on the user remote station. The image analysis in pathology requires uploading the image to perform the analysis. In this paper the practical realization of image processing in pathology at using Matlab functions by JSP and Tomcat server [13] for is presented.

## Implementation

In this study the author proposes to build the remote platform for image analysis based on Matlab function, JSP and Tomcat server. These tools with additional database provide all functions required to realize the task. The basic requirements of the programs are as follows:

• *Java* Runtime Environment v. 6 or higher

• Tomcat 6.0.20 or higher

• Matlab 7.9 or higher

• Matlab Toolboxes: MATLAB Compiler v. 4.11, MATLAB Builder JA v. 2.0.4, Database Toolbox v. 3.6 and Image Processing Toolbox v. 6.4.or higher.

**Figure 1:**
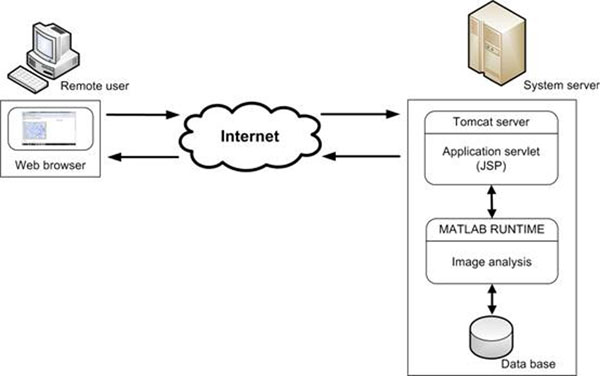
The main scheme of remote image analysis system

The main scheme of the platform containing Tomcat server as a container of system’s servlet, the servlet itself written in JSP language, Matlab and data base is presented on the fig. 1. The remote user with the help of web browser connects with the system server via internet. Any web explorer, such as Mozilla Firefox, Internet Explorer etc. can be used. The Tomcat server is a provider of internet service and works as a servlet container. The web page viewed by the remote user is generated by the application servlet written in JSP language. This servlet realizes response and sends any data from web browser, connecting with Matlab Runtime Library [7] for running image analysis functions. The storage of any data in the data base is controlled by Matlab functions. This data base can be situated in the system server or in a other computer, connected with server e.g. via LAN. The numerical and graphical results of image analysis are returned from Matlab functions to the application servlet and transmitted via internet to web browser. All tools of image analysis platform can be run on the system server and any operation performed on the data can be realized on this computer. The alternative approach with data base placed on the other computer connected with server e.g. via LAN can be also applied.

The proposed remote system requires the set of specific tools and methods for efficient work in practical application. The main task to solve is the realization of the image uploading and returning the results by remote user, working as one of many users of the system. The appropriate mechanisms for realization of these features of the remote platform are implemented by JSP servlet. The first step done by the user is uploading the image for the analysis. The *multipart/form-data* data form is one of the most commonly used for submitting files via HTTP. This form offers possibility of uploading image file to the remote post frame. The simple code can be written as

*<form name=”PlatformName” action=”System.jsp” method=”post” ENCTYPE=”multipart/form-data” >*.

The second step is to send the request from client to server. This action requires implementation of acquiring the image file and transmitting it via internet to JSP servlet. The interpretation of the data can be performed by Matlab function. To make such transmission the image file must be read from multipart request via byte stream and used as a parameter for the Matlab function. The sample code of file reading via stream is as bellow

tmpFile = multipartRequest.getFile(“upload”);

FileInputStream fis = new FileInputStream(multipartRequest.getFile(“upload”));

byte dataBytes[] = new byte[fis.available()];

int byteRead = 0;

int wsk=1;

while (wsk>-1) {

byteRead = fis.read(dataBytes);

wsk=byteRead;

*}*.

The Matlab function can be run by command

Object[] result =

componentObject.function_name(

1, // Always one -- a structure array

**Figure 2:**
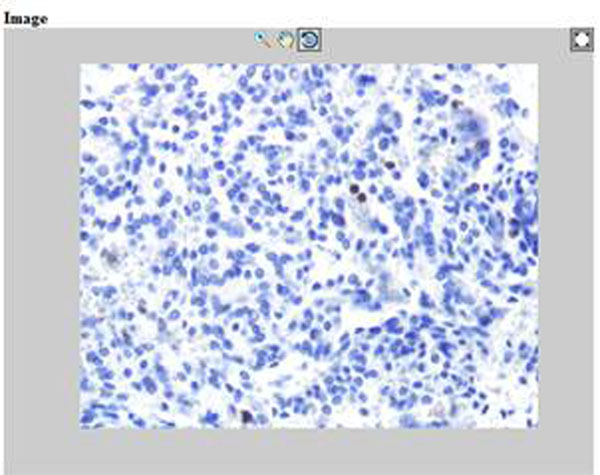
The example of the Matlab web figure

dataBytes,

other parameters….);

returning the results of the executing this function in a *result* object. The *function_name* is a name of Matlab function and *componentObject* is the object created by servlet, containing Matlab functions as object methods. The *result* object may contain any numerical data, such as file name, size and other, and also Matlab web-figure. This web-figure is a virtual type of classical Matlab figure obtained at the stationary use of application and can be placed in HTML code. This web-figure is equipped with zoom, rotate and move buttons, as shown in Fig. 2. In the presented solution the remote image analysis is realized by different Matlab algorithms. These algorithms should be compiled to executable *jar* file with the help of Matlab Compiler and Matlab Builder JA toolbox. The Matlab function must be declared with set of input data and output structure containing the numerical results and Matlab web-figure. Any function prepared in that manner can be used as a Java function in Java Servlet Pages (JSP). The graphical user interface providing the input data and displaying the results (also in graphical form) must be implemented in JSP.

The JSP servlet realizes the process of uploading the input data (image and description data), transforming the data from html to Matlab function(s) and back to html and providing any commands initiated by the user. The Matlab functions must be compiled into jar file and also added to Tomcat system servlet.

The JSP servlet can initiate the Matlab functions. These functions are interpreted by *Matlab* Component *Runtime* (MCR) Library that must be installed in the system’s server. The second way (more recommended) is installing full Matlab program on the server. Indeed any Matlab functions compiled into jar file are executed by the Matlab library. The designed Matlab function(s) can realize image processing by using any *Image processing toolbox*[7] tools and connecting with the data base for storage of the image(s) with the help of *Database toolbox*[*7*]. The data base can be used for storage of the data description and results, and also for creating the unique name/number for the image file/case number. It is possible also using any other useful Matlab tools, such as neural networks as classifiers or statistic analyzers [7]. In the presented solution Matlab can offer many tools without necessity to implement in the other language for remote using.

The very important problem in programming of a remote platform for image analysis is parallel computing. The data provided by the user should be visible only by owner and not by the other users. The practical solution of this requirement can be realized by initializing the sessions. Any data and image uploaded by the user, as well as the results of image analysis should be stored only in a specific session initiated only for this user. The setting and getting of the selected attributes can be realized by the sample code

session.setAttribute( " AttribureName ","NewValue")

session.getAttribute("AttribureName").

The same way we use Matlab web-figure. For HTML code generation in the JSP servlet can use command

**Figure 3:**
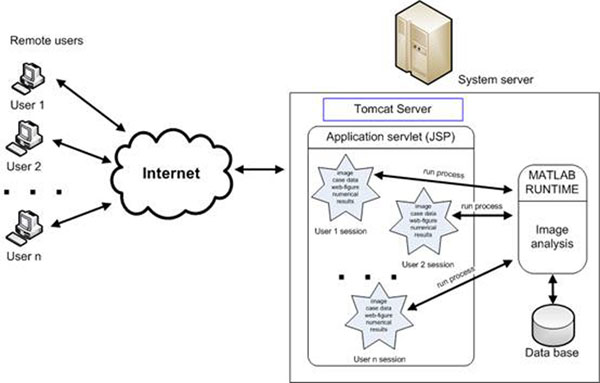
The scheme of the parallel computing in the remote platform

<wf:web-figure name="myFigure" scope="session" root="WebFigures"></wf:web-figure>

where scope parameter controls the process of getting the web-figure from the user session. The scheme of the parallel computation on the remote platform is presented in the fig. 3.

The final problem is to provide the synchronization of the connection to data base when it is necessary. This case appears when the user’s session adding the new case to the database, requesting the unique number for it. At any time only one connection channel can be open and still will be closed any operation must be thread-safe by using *synchronized* command.

## Results

The proposed tool for remote image analysis was tested by using the Computerized Analysis of Medical Images (CAMI) software developed by author. The user provides image and case information (diagnosis, staining, image parameter etc.) and when analysis is initialized, the input data and image are sent to servlet on Tomcat. There exist two cases of connection with servlet. The first one is provided for image upload and viewed in Matlab web-figure. The second one is for uploading the image (if it wasn’t upload earlier) as well as running the image analysis. When analysis is done, client obtains the results in the form of annotated image with marked recognized cells and quantitative measures. Additionally, the results are stored in server database.

One of the practical question is how to store images in the data base. In CAMI realization the image file is saved in the fixed directory on the server. When image is uploaded only for viewing it, the Matlab function generates 10 digits random number used as a file name and returns it to the user session. When analysis is run, the image processing function gets this image name as well as case description data. The program performs image analysis and when it was successfully done the case will be added to the data base. The function initiates the connection with the data base, adds this new case together with the results of analysis and obtains the new unique etiquette for the processed image. Subsequently, the connection is lost, 10 digits name of the image file is changed into the new unique name and finally web-figure and numerical results are returned to the appropriate user session. After finishing the analysis, the servlet generates HTML code with results. This code is transmitted to the user web browser and the results of image analysis are shown.

The internet platform was tested on PC Intel Core2 Duo T9600 2.8GHz 4GB RAM server with 768x576 pixel size, 1.28Mb tiff format images with meningioma tumour (x400, Ki-67/MIB-1). The consumption time was as follow: for analysis by CAMI locally on server – 3.5 seconds, for remote analysis – 26 seconds which covers 22 seconds for data transfer via internet connection. For jpg format image (768x576 pixel size, 102 Kb) the consumption time was reduced to 14 seconds. The system can analyze any image formats recognized by Matlab *imread* function. The user connection speed was 2 Mb/s for download and 0.5 Mb/s for upload.

## Discussion

The pathological image analysis is a one of the possible specific direction of medical image processing. The considered images represent practically infinite diversity regarding the types of a tissue or likewise types of staining. This differences are the reason of developing many methods and approaches to the computerized image analysis.

Currently we can find many algorithms and languages used for analysis of these images. One of the most advanced tools in the image analysis is Matlab with Image processing toolbox. This program offers many functions realizing very useful operations, forming the advanced mathematical tools. For scientists involved in research of image processing it is a very popular language allowing the implementation and development of many advanced approaches to the image analysis. However, the remote image processing based on Matlab functions via internet is still in early stage of development. One example of image analysis by Matlab with web browser can be found in the paper [12]. Unfortunately, this example works only with the image files stored on server, not with the images provided by user from the remote computer. This solution provides no tools for data storage in data base and parallel computing.

In this paper the approach based on Tomcat server, JSP servlet and connection to data base directly from the Matlab function is proposed. The practical realization of this approach requires finding many keys and solving some problems for offering the practical and useful tool. The presented realization allows uploading the image file from remote computer to servlet and Matlab platform, the connection to data base for data storing and mechanism of parallel computing. To offer the platform for many users together, the session, as a user data container, was applied.

The other significant feature of the proposed solution is that the processes of image analysis and stored data aren’t visible for remote users. This provides high security of the proposed system and makes the system scalable. The data base can be hidden in the local LAN network and a single process of image analysis can be also moved to the other computers/cores.

## Conclusions

The results have confirmed that designed remote platform can be useful in pathology image analysis. The platform is easy to be implemented with the help of Matlab and free programs, such as Java and Tomcat. The data base can be also built using any other popular software e.g. Oracle, MS Access, SQL etc. and runs on the ODBC protocol in MS Windows.

The consumption time depends mainly on the image size and speed of internet connections. The presented implementation can be used for many types of analysis (different staining, tissue, morphometry approaches etc.). The significant question is implementation of the JSP page in the multisession form, that can be used parallel by many users. The presented platform for image analysis in pathology can be especially useful for laboratory having no image analysis system.

## Availability and requirements

• Project name: Computerized Analysis of Medical Images

• Project home page: http://www.cami.iem.pw.edu.pl

• Operating system(s): MS Windows

• Programming language: Matlab, Java, Java Servlet Pages

• Other requirements: Java Runtime Environment 6 or higher, Tomcat 6.0.20 or higher, Matlab 7.9 or higher, Matlab Toolboxes: MATLAB Compiler 4.11, MATLAB Builder JA 2.0.4, Database Toolbox 3.6 and Image Processing Toolbox 6.4.or higher

• License: Matlab license with depicted toolboxes, Java and Tomcat – GNU licence.

• Any restrictions to use by non-academics: licence needed.

## Competing interests

The authors declare that they have no competing interests.

## Authors' contributions

TM – 100 % contribution of the presented paper.
